# Triple Calamity in a Smoker: Idiopathic Pulmonary Fibrosis, Chronic Pulmonary Aspergillosis, and Lung Adenocarcinoma

**DOI:** 10.7759/cureus.102539

**Published:** 2026-01-29

**Authors:** Subham Sahoo, Mahismita Patro, Prasanta R Mohapatra, Mukund Sable, Vinay Kumar Hallur, Manoj K Panigrahi

**Affiliations:** 1 Department of Pulmonary Medicine and Critical Care, All India Institute of Medical Science, Bhubaneswar, Bhubaneswar, IND; 2 Department of Pulmonary Medicine and Critical Care, All India Institute of Medical Sciences, Bhubaneswar, Bhubaneswar, IND; 3 Department of Pathology and Laboratory Medicine, All India Institute of Medical Sciences, Bhubaneswar, Bhubaneswar, IND; 4 Department of Microbiology, All India Institute of Medical Sciences, Bhubaneswar, Bhubaneswar, IND

**Keywords:** chronic pulmonary aspergillosis, idiopathic pulmonary fibrosis, interstitial lung disease (ild), lung adenocarcinoma, pulmonary aspergillosis

## Abstract

We report, to the best of our knowledge, the first case of idiopathic pulmonary fibrosis (IPF) with concomitant chronic pulmonary aspergillosis (CPA) and lung adenocarcinoma in a 62-year-old male smoker. The patient presented with a dry cough for three years and intermittent hemoptysis for the last year. The radiological evaluation suggested IPF and bilateral lung masses with an air-crescent sign, which progressively increased in size. Subsequent investigations, including bronchoscopy and transthoracic biopsy, confirmed the presence of the triple calamity of IPF, CPA, and lung cancer. He was managed with antifungal, antifibrotic, and systemic chemotherapy following a multidisciplinary discussion. This rare coexistence highlights the complex diagnostic and therapeutic challenges in patients with IPF presenting with new cavitary or progressive lesions.

## Introduction

Idiopathic pulmonary fibrosis (IPF) predisposes patients to both chronic infections and malignancy due to architectural distortion and chronic inflammation of the lung parenchyma. Distinguishing infection from malignancy in IPF represents a significant clinical challenge owing to similar radiologic findings and shared symptoms [1]. There are scarce reports of the coexistence of chronic pulmonary aspergillosis (CPA) as well as primary lung cancer in IPF [2-4]. Also, lung cancer has been reported to be associated with pre-existing fibro-cavitary disease, old tuberculosis, or rarely CPA [5]. To the best of our knowledge, we report the coexistence of IPF, CPA, and lung cancer for the first time in the literature.

## Case presentation

A 62-year-old male, a farmer by occupation, a current smoker with a 40-pack-year history, presented with chief complaints of dry cough for three years and intermittent scanty hemoptysis for the last year. His prior evaluation outside revealed largely unremarkable routine blood investigations except for mildly elevated erythrocyte sedimentation rate and C-reactive protein (Table 1).

**Table 1:** Baseline investigations of the patient at presentation ESR: erythrocyte sedimentation rate; AST: aspartate transaminase; ALT: alanine transaminase

Investigation	Patient Value	Normal Range
Hemoglobin (g/dL)	13.4	13–17
Total Leukocyte Count (cells/mm³)	7,800	4,000–11,000
Platelet Count (×10⁹/L)	240	150–450
ESR (mm/hr)	28	<20
C-Reactive Protein (mg/L)	6.2	<5
Serum Urea (mg/dL)	36	10–45
Serum Creatinine (mg/dL)	0.9	0.6–1.2
Total Bilirubin (mg/dL)	0.8	0.1–1.2
Direct Bilirubin (mg/dL)	0.5	0.0–0.3
AST (U/L)	32	<40
ALT (U/L)	29	<40
HbA1c (%)	5.4	4.0–5.6
Total IgE (IU/mL)	420	<100

**Figure 1:**
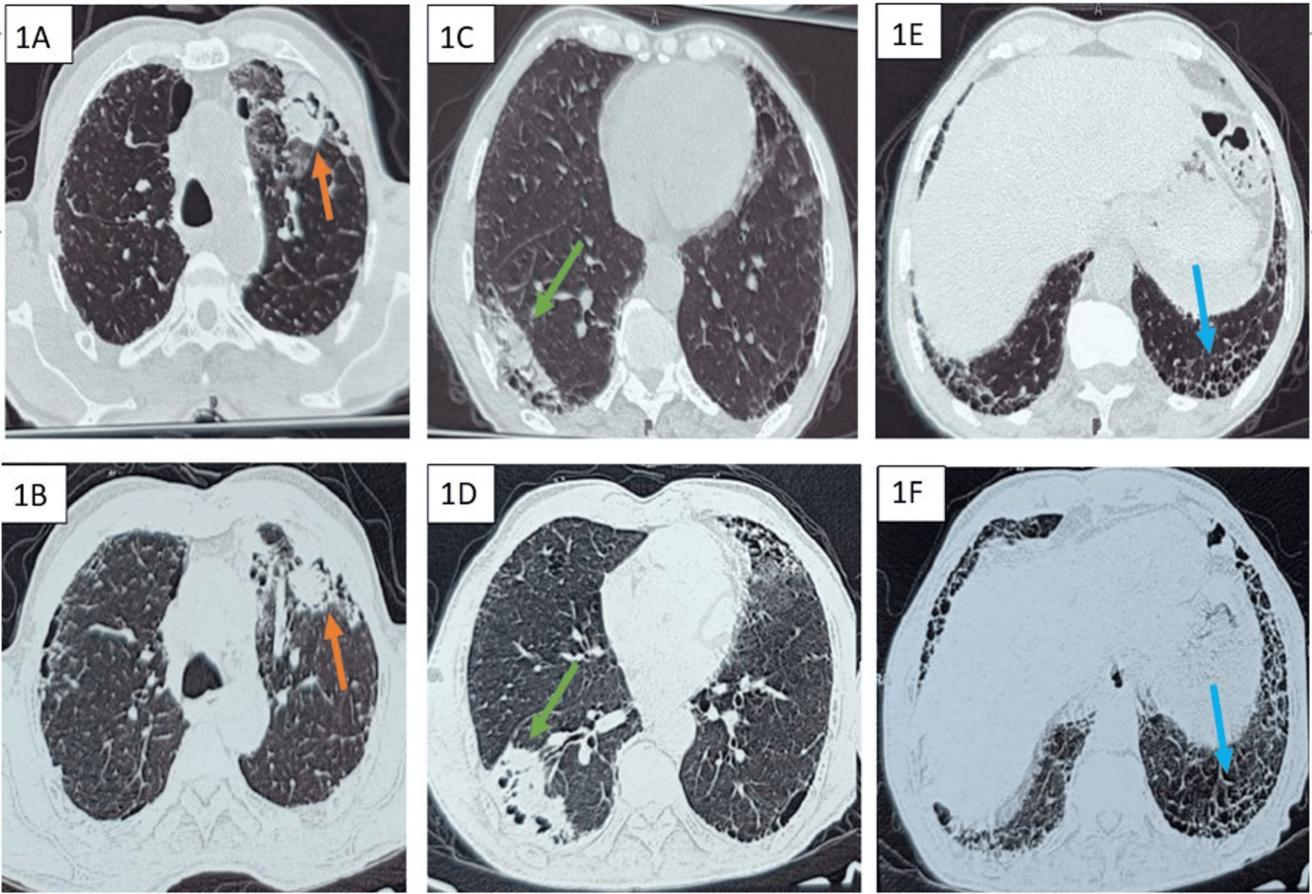
High-resolution computed tomography images of the patient The upper panel displays initial findings, and the lower panel shows follow-up images after three months. 1A & 1B: Cross sections at the level of the lower trachea show a left upper lobe mass-like lesion with an air crescent sign (arrow) and progressive increase in fibrosis. 1C-1D: Cross-sections at the level of cardiac chambers suggestive of a right lower lobe mass-like lesion (arrow) with progressive increase in size and subpleural fibrosis. 1E & 1F: Cross-sections at basal levels show bilateral subpleural septal thickening and honeycombing, suggestive of a definite usual interstitial pneumonia pattern (arrow).

He had undergone thoracic imaging twice, at a gap of three months. The first high-resolution CT (HRCT) of the thorax showed a definite usual interstitial pneumonia pattern suggestive of IPF, along with mass-like opacities with an air crescent sign in the right lower lobe and left upper lobe (Figure 1). Based on the initial radiology, with a suspicion of CPA, the patient was started on itraconazole by the previous doctor. Due to the persistence of respiratory symptoms, including hemoptysis, a repeat HRCT of the thorax was performed three months later. The latest scan revealed a progressive increase in the fibrosis of the bilateral lung fields, accompanied by an enlargement of the right lower lobe mass lesion (Figure 1).

**Figure 2:**
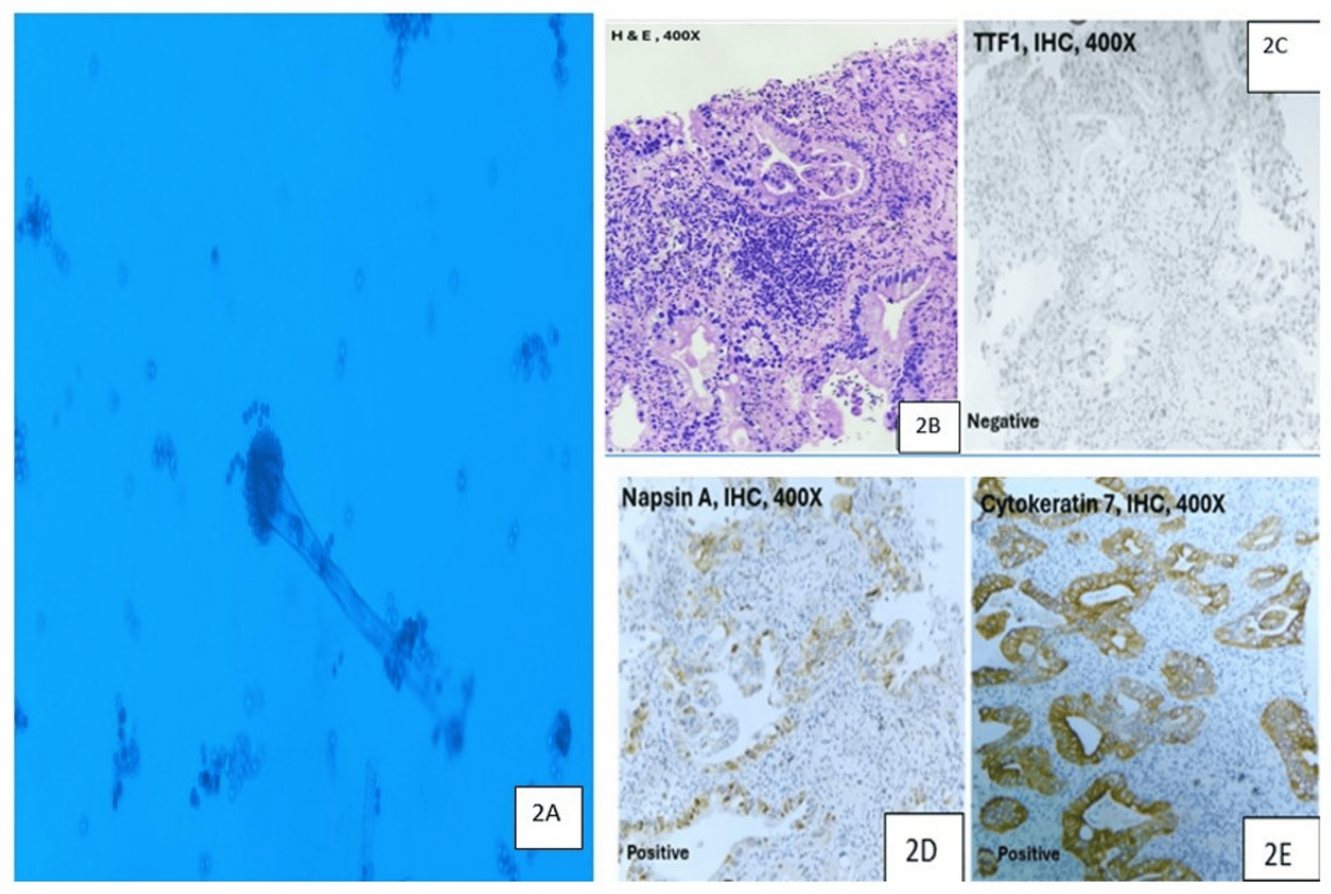
2A: Bronchoalveolar lavage (BAL) fluid fungal culture on inhibitory mold agar exhibited septate, hyaline, acute-angle dichotomous branching hyphae characteristic of Aspergillus fumigatus. 2B-2E: Histopathological examination findings of biopsy of right lower lobe mass. 2B: Haematoxylin and eosin–stained section (400×) showing tumor cells arranged in glandular and cribriform patterns with vacuolated cytoplasm and moderate nuclear pleomorphism, consistent with adenocarcinoma. 2C-2E: Immunohistochemical panel showing tumor cells positive for napsin A and cytokeratin 7 (CK7) and negative for thyroid transcription factor-1 (TTF-1). IHC: immunohistochemistry

Immunological work-up showed positive *Aspergillus*-specific IgE at 88.47 kU/L (reference: <0.7), and *Aspergillus*-specific IgG was negative at 5.78 U/mL (reference range 0-8) using the enzyme-linked immunosorbent assay method. A rapid test using immunochromatography, specifically a lateral flow assay, for *Aspergillus*-specific IgM and IgG antibodies was positive. Bronchoscopy showed a normal tracheobronchial tree, and bronchoalveolar lavage (BAL) fluid was taken from the left upper lobe. His sputum and BAL fluid evaluation for malignant cytology, bacteria, and mycobacteria were negative. BAL fluid potassium hydroxide (KOH) mount showed thin hyaline septate hyphae. BAL fluid fungal culture on inhibitory mold agar confirmed growth of Aspergillus fumigatus on day 7 of incubation at both 25°C and 37°C (Figure 2A). Based on clinical, radiological, immunological, and microbiological (CRIM) criteria, a diagnosis of CPA was made in the background of IPF [6,7]. Itraconazole 200 mg twice daily and nintedanib 150 mg twice daily were started. A CT-guided biopsy of the right lower lobe mass was done to rule out malignancy. It showed tumor cells arranged predominantly in glandular and cribriform patterns with immunopositivity for napsin A and cytokeratin 7 (CK7), while being immunonegative for thyroid transcription factor-1 (TTF-1) and cytokeratin 20 (CK20) (Figures 2B-2E). The immunohistochemistry profile supported a diagnosis of primary adenocarcinoma of lung origin. Thus, a confirmatory diagnosis of the triple calamity of IPF, CPA, and lung adenocarcinoma was made. 

**Figure 3:**
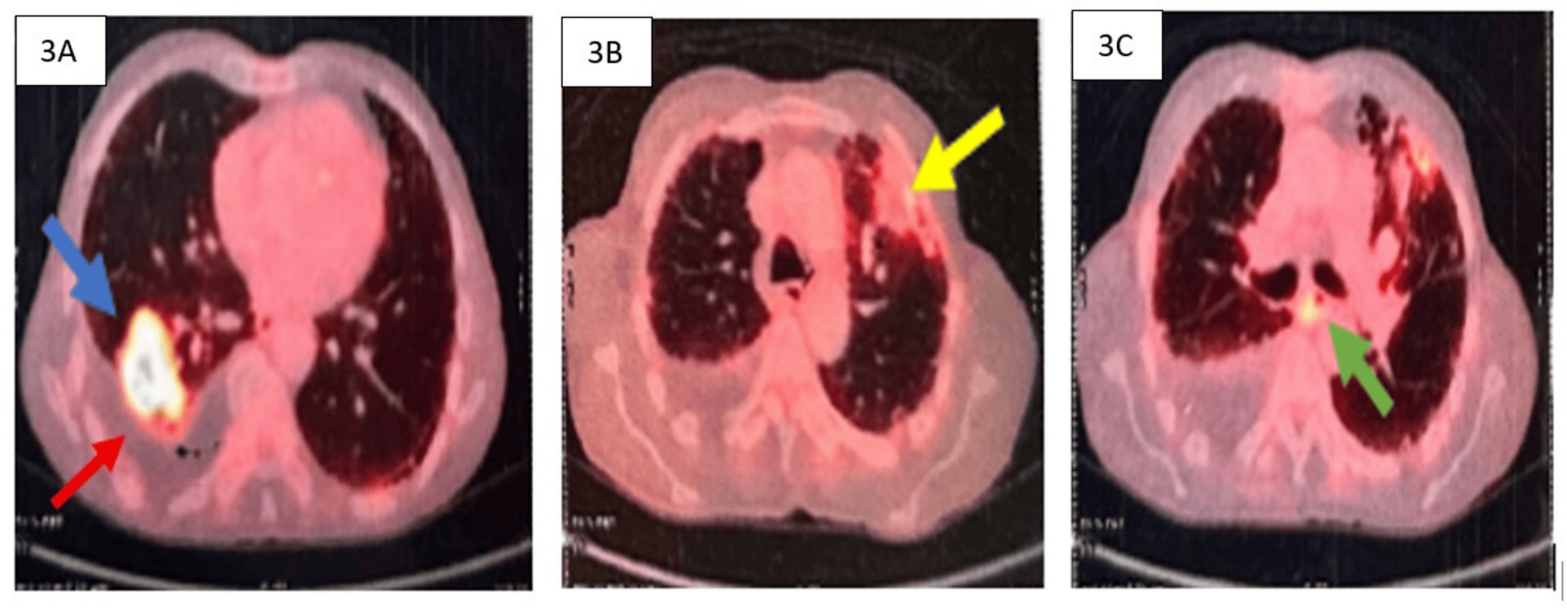
Findings of an 18-fluorodeoxyglucose (FDG) whole-body PET-CT scan. 3A: FDG-avid (maximum standardized uptake value (SUVmax) 11.7) costal pleural-based necrotic mass lesion in the superior and lateral basal segments of the right lower lobe (blue arrow) and associated pleural effusion. 3B: FDG-avid (SUVmax 4.7) pleural-based necrotic nodule in the anterior segment of the left upper lobe (yellow arrow). 3C: FDG-avid mediastinal lymph nodes.

A whole-body 18-fluorine fluorodeoxyglucose (FDG) PET-CT scan was done for staging and metastatic evaluation, which showed an FDG-avid costal pleural-based cavitary lesion involving the superior and lateral basal segments of the right lower lobe of size 4.5 cm x 2.9 cm x 6.3 cm (maximum standardized uptake value (SUVmax) 11.7) with surrounding fibrosis. Additionally, FDG avidity was noted in the left upper lobe mass lesion (SUVmax 4.7) and multiple mediastinal lymph nodes suggestive of metastatic involvement. A non-FDG-avid moderate right pleural effusion was also observed. There was no evidence of clinically significant abnormal FDG uptake elsewhere (Figure 3). Contrast-enhanced MRI of the brain revealed no abnormalities. The histological confirmation of malignancy in the left upper lobe mass lesion or the new onset of right pleural effusion could not be done owing to high procedural risk and patient refusal.

Further management options were discussed in the institutional tumor board. The underlying pulmonary fibrosis and CPA posed a high risk of complications for a repeat biopsy of the left lung lesion or surgical excision of the right lower lobe mass. The patient’s biopsy specimen, subjected to molecular profiling for available targetable mutations and immunotherapy markers, including epidermal growth factor receptor (EGFR), anaplastic lymphoma kinase (ALK), c-ros oncogene 1 (ROS1), and programmed death-ligand 1 (PD-L1), was negative. Hence, it was decided to start treatment with standard chemotherapy for stage IV adenocarcinoma of the lung while continuing medical management for CPA and IPF with close clinical monitoring [8]. The patient was given combination chemotherapy with pemetrexed and carboplatin every 21 days, planned for four cycles followed by maintenance pemetrexed based on response assessment. Simultaneously, he continued on nintedanib 150mg twice daily for IPF and itraconazole 200mg twice daily for CPA, with monitoring for adverse effects. 

## Discussion

The coexistence of IPF, CPA, and primary lung malignancy in the same patient represents a rare and diagnostically challenging clinical scenario. The underlying pathogenetic mechanisms of this co-existence are poorly understood.

CPA is typically seen in pre-existing structural lung disease, such as post tuberculosis, with rare reports in IPF. The pathogenesis of IPF-related CPA is thought to involve fibrotic architectural distortion, accompanied by honeycombing and traction bronchiectasis, which creates cystic spaces that predispose to *Aspergillus *colonization [3,4,9]. The absence of a history of prior tuberculosis in our patient, with HRCT findings of honeycombing, suggests IPF itself could be the underlying substrate for fungal colonization.

IPF patients have a markedly increased risk of lung cancer, with reported prevalence varying from 4% to 48% [1,2]. Proposed mechanisms include chronic epithelial injury, shared genetic/epigenetic changes (e.g., p53 mutations, DNA hypermethylation), and a tumor-promoting fibrotic microenvironment [9]. Smoking, present in our patient, probably acted synergistically with fibrotic injury to accelerate malignant transformation. While squamous cell carcinoma is more common in IPF, adenocarcinoma, as in our case, is also reported, especially in peripheral fibrotic zones.

CPA and lung cancer often mimic each other radiologically, but they can also rarely coexist. Cavitary carcinomas, particularly the squamous cell types, may exhibit fungal colonization. Lung cancer may arise within fibrotic or CPA-involved areas, where chronic inflammation and hypoxia further drive oncogenesis. Imaging alone is unreliable in differentiating them, thus necessitating tissue diagnosis [5]. In our case, the fibrotic masses were diagnosed as CPA based on standard criteria. The diagnosis of CPA requires the presence of clinical criteria (symptoms of cough, dyspnoea, hemoptysis, weight loss, etc. over three months), radiologic evidence (cavitary or fibrocavitary lesions with or without fungal balls), and any one or both of the immunological (*Aspergillus*-specific IgG positive) or microbiological (direct evidence of *Aspergillus *on sputum or BAL fluid fungal stain or culture) criteria. Our patient satisfied the clinical and radiologic criteria along with microbiologic evidence, although the immunologic criteria were negative. Hence, suspected CPA patients should undergo sputum or BAL fluid fungal examination for a confirmatory diagnosis when immunological evidence for *Aspergillus *is negative. In our patient, underlying adenocarcinoma was confirmed on tissue biopsy, underscoring the necessity of histopathological confirmation in atypical or progressive lesions.

This coexistence is likely to complicate management with multiple drug-drug interactions of chemotherapy, antifibrotics, and antifungals. This also has a high surgical risk, even if the cancer is resectable. The prognosis is also poor, as each condition independently carries high mortality. Due to the rarity of the case, a multidisciplinary approach integrating pulmonology, oncology, mycology, and radiology should be considered for optimal management.

## Conclusions

The coexistence of IPF, CPA, and lung cancer, though rare, is plausible due to shared risk factors and overlapping pathophysiological pathways. Hence, routine surveillance imaging in smokers with IPF is needed to detect superimposed infections and malignancies early. There should be a low threshold for biopsy of atypical or progressive lesions in IPF.
